# Research priorities to enhance life for people with spinal cord injury: a Swedish priority setting partnership

**DOI:** 10.1038/s41393-023-00913-2

**Published:** 2023-07-20

**Authors:** Jeanette Melin, Emelie Axwalter, Gunilla Åhrén, Katharina S. Sunnerhagen, Åsa Lundgren-Nilsson, Johanna Wangdell

**Affiliations:** 1 Gothenburg Competence Centre for Spinal Cord Injury, University of Gothenburg and Sahlgrenska University Hospital, Gothenburg, Sweden; 2 Division Safety and Transport, Department Measurement Science and Technology, RISE Research Institutes of Sweden, Gothenburg, Sweden; 3 Department of Clinical Neuroscience, Sahlgrenska Academy, Institute of Neuroscience and Physiology, Gothenburg, Sweden; 4 Department of Neurocare, Sahlgrenska University Hospital, Gothenburg, Sweden; 5 Department of Hand Surgery, Institute of Clinical Sciences, University of Gothenburg, Gothenburg, Sweden; 6 Centre for Advanced Reconstruction of Extremities, Sahlgrenska University Hospital/Mölndal, Gothenburg, Sweden

**Keywords:** Health policy, Spinal cord diseases

## Abstract

**Study design:**

Mixed-method consensus development project.

**Objective:**

To identify the top ten research priorities for spinal cord injury (SCI).

**Setting:**

Nationwide in Sweden in 2021–22.

**Methods:**

The PSP process proposed by the James Lind Alliance was used. It comprises two main phases: question identification and priority selection. People living with SCI, relatives of people with SCI as well as health professionals and personal care assistants working with people with SCI were included.

**Results:**

In the first phase, 242 respondents provided 431 inputs addressing potentially unanswered questions. Of these, 128 were beyond the scope of this study. The remaining 303 were merged to formulate 57 questions. The literature review found one question answered, so 56 questions proceeded to the prioritisation. In the second phase, the interim prioritisation survey, 276 respondents ranked the 56 questions. The top 24 questions then proceeded to the final prioritisation workshop, at which 23 participants agreed on the top ten priorities.

**Conclusions:**

This paper reveals issues that people living with SCI, relatives of people with SCI as well as health professionals and personal care assistants working with people with SCI find difficult to get answered. The top-priority questions for people living with SCI in Sweden concern specialist SCI care and rehabilitation, followed by a number of questions addressing physical health. Other topics, from the 56 key questions include Mental health, Ageing with SCI, Community support and personal care assistance, and Body functions. This result can guide researchers to design appropriate studies relevant to people with SCI.

**Sponsorship:**

The project was funded by the Gothenburg Competence Centre for Spinal Cord Injury and the Swedish Association for Survivors of Accident and Injury (RTP).

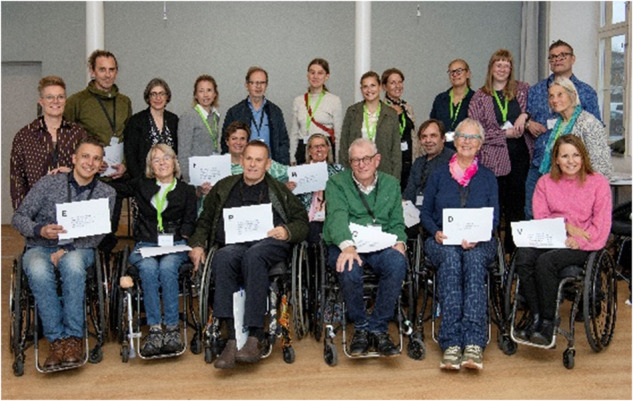

## Introduction

Spinal cord injury (SCI) affects many individuals worldwide. The annual incidence of traumatic SCI is estimated to range from 12.1 to 57.8 per million worldwide [1], while the estimate for non-traumatic SCI, in the absence of high-quality data, is unreliable but may be up to 80 per million [2]. Furthermore, there is substantial variation in mortality and longevity in the SCI population, compared with the general population, among WHO regions and according to countries’ income levels [3]. The magnitude of the injury and its repercussions vary greatly from one individual to another. For decades it has been well established that surviving SCI is not all that matters. Rather, rehabilitation must focus on people living with SCI, and providing support to enhance their lives. Another key goal of rehabilitation after SCI is to enable them to achieve a satisfactory quality of life [4–8].

A spinal cord injury research strategy for Australia and New Zealand has recently been developed. Its aims are to facilitate collaborative and multicentre research; ensure consistency among sites in terms of patient selection, treatment and evaluation; and, in turn, enhance neuroprotection and functional recovery, reduce the impact of secondary complications and underpin clinical best practice and policy to improve health, participation and quality of life [9–11]. One of the four areas in the research strategy includes boosting consumer engagement in research, while the other three mainly focus on research capacities [11]. The research strategy for Australia and New Zealand did, however, not focus on specific research questions and priorities to be addressed.

In response to a research agenda based on the issues that matter most to those who need to use the research in their daily lives—that is, in the context, people living with SCI—the James Lind Alliance has developed priority setting partnership (PSP) methodology [12]. The PSP comprises two main phases: identification of uncertainties and a process of prioritisation. Through the PSP, the uncertainties identified become priority research questions, as described below under “Methods”.

In 2013–14, a PSP identifying the top ten research priorities for SCI was conducted in the UK [13]. It focused on identifying priorities related to care and treatment (specifically, asking *What question[s] would you like researchers to answer that will help improve the treatment and care of people living with SCI?*). However, a rapid review conducted in 2015 stressed research considerations pertaining to perspectives on quality of life among people living with SCI [9]. Thus, there is a need to move research outside hospitals and include the importance of resilience, family sustainability, depression and motivation in how well these people cope with day-to-day living and maintain their health, well-being and quality of life in the long term [6, 7, 10]. It is not likely that every country or region should have their own research agenda, but with different health care and social security systems, one must investigate priorities in other places also. The same holds for investigation of potential new research priorities over time.

A Swedish needs-assessment project, combining the PSP methodology and workshops, has therefore been under way in 2021–22. The overall aim of the project is to identify needs and key questions to enhance life for people living with SCI in Sweden, as perceived by these individuals themselves and their relatives, as well as health professionals and personal care assistants^1^ working with people living with SCI. The project is in three parts: (a) needs to be met; (b) questions to be answered; and (c) knowledge required. In this study, we address part (b) and present the top ten research priorities identified through the PSP.

## Methods

**Fig. 1:**
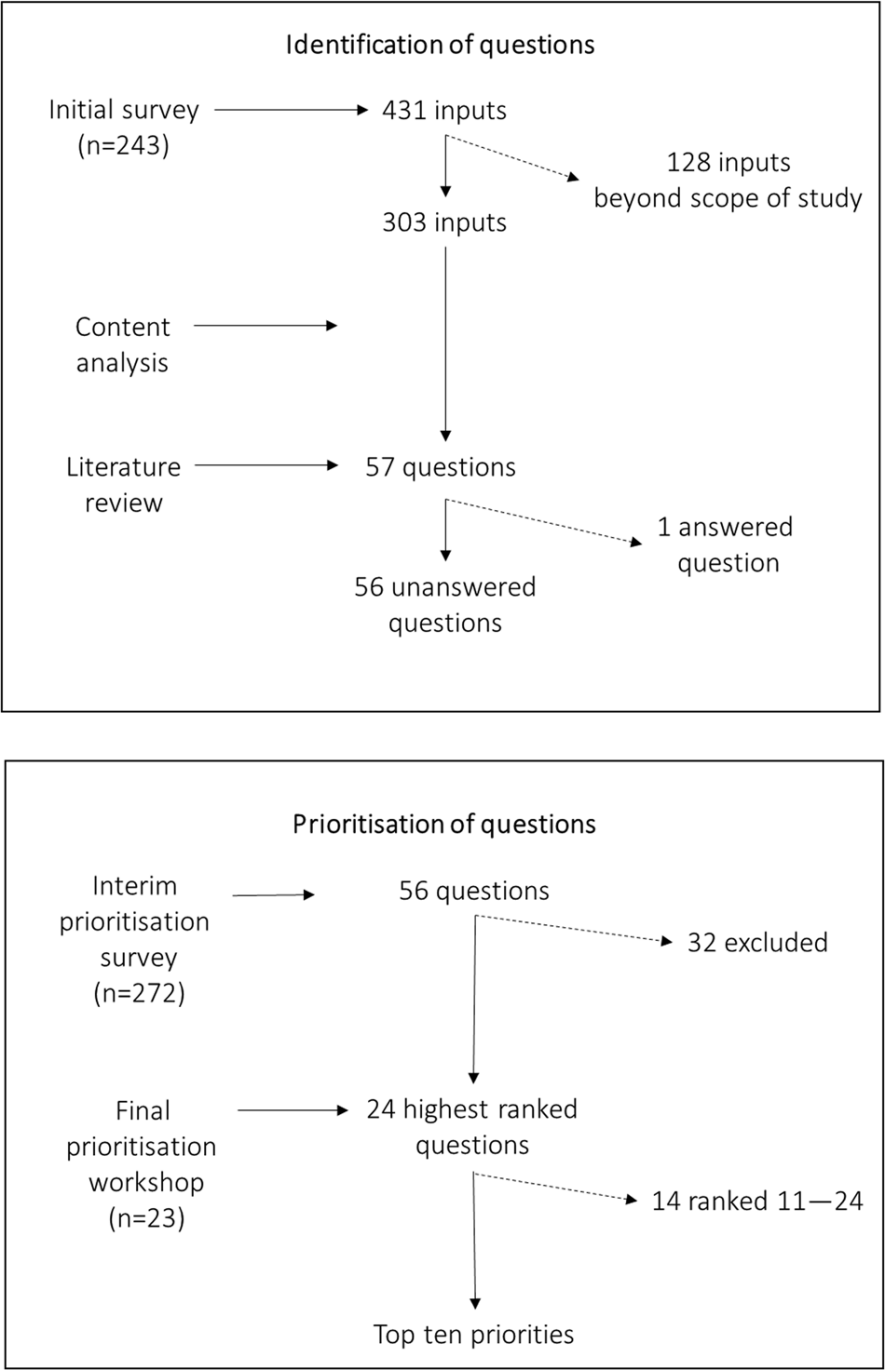
A simplified flow chart of the JLA method: how inputs (such as statements and issues) from the initial survey were then processed to form questions and the top ten priorities were derived.

In this study we followed the overall PSP process described in detail in the *JLA Guidebook* [15]. The PSP comprised two main phases: identification of questions and a prioritisation process. Figure 1 shows a simplified flow chart of the JLA method, illustrating how inputs (such as statements and issues) from the initial survey were processed to form questions and thereafter the top ten priorities defined in the present study.

Throughout the PSP process, a JLA advisor and an expert group have assisted the research team. The role of the 10-strong expert group was to advise on appropriate language, methods and inclusive engagement strategies to reach a diverse range of people living with SCI, relatives of people with SCI as well as health professionals and personal care assistants working with people with SCI were included.

### Identification of uncertainties

An online survey using Microsoft Forms was conducted in November and December 2021 among people living with SCI, relatives of people with SCI as well as health professionals and personal care assistants working with people with SCI were included. Respondents were recruited on social media, at clinics and in networks all over Sweden. The survey took 5 weeks. After 3 weeks, we checked the respondents’ characteristics for representativeness; this resulted in extra efforts to reach men with SCI, people with high-level SCI and Stockholmers.

In total, 243 people responded to the survey. Table 1 summarises their personal particulars. They were asked to state one to five questions that themselves or others find difficult to find answers to.

**Table 1:** Personal characteristics of respondents in the initial survey.

	People living with SCI	Relatives	Health professionals	Personal care assistants	Total
	*n* = 128 (53)	*n* = 34 (1)	*n* = 78 (32)	*n* = 3 (1)	*n* = 243
Gender					
Male	70 (55)	6 (18)	15 (19)	0	91 (38)
Female	58 (45)	28 (82)	62 (81)	3 (100)	151 (62)
* Missing*	–	–	1	–	1
Age, years					
Mean (SD)	54 (11)	54 (12)	44(12)	36 (8)	51 (13)
Median (range)	55 (28–78)	54 (28–72)	45.5 (22–68)	33 (30–45)	51 (22–78)
<30	2 (2)	2 (1)	13 (18)	1 (33)	18 (8)
31–45	25 (20)	5 (17)	24 (32)	2 (66)	57 (24)
46–60	62 (50)	15 (50)	30 (41)	–	107 (45)
60–75	34 (27)	8 (27)	7 (9)	–	49 (21)
>75	2 (2)	–	–	–	–
* Missing*	3	4	–	–	7
Municipality group					
Large cities and nearby municipalities	54 (46)	16 (48)	28 (39)	1 (33)	99 (44)
Medium-sized towns and nearby municipalities	46 (39)	13 (39)	26 (36)	1 (33)	86 (38)
Small towns/urban areas and rural municipalities	18 (15)	4 (12)	18 (9)	1 (33)	41 (18)
* Missing*	10	1	6	–	16
Time post injury (TPI)					
Mean (SD)	19 (15)	–	–	–	–
Median (range)	16 (0–55)	–	–	–	–
1–5	26 (21)	–	–	–	–
6–10	17 (14)	–	–	–	–
11–15	18 (15)	–	–	–	–
16–20	12 (10)	–	–	–	–
21–25	5 (4)	–	–	–	–
26–30	8 (7)	–	–	–	–
>30	37 (30)	–	–	–	–
* Missing*	5	–	–	–	–
Cause of injury					
Traumatic	97 (80)	–	–	–	–
Non-traumatic	24 (20)	–	–	–	–
* Missing*	7	–	–	–	–
Type of injury					
Complete	55 (45)	–	–	–	–
Incomplete	67 (55)	–	–	–	–
* Missing*	6	–	–	–	–
Tetraplegia	61 (49)	–	–	–	–
Paraplegia	63 (51)	–	–	–	–
* Missing*	4	–	–	–	–
Relative					
Partner	–	18 (53)	–	–	–
Parent	–	12 (35)	–	–	–
Sibling	–	2 (6)	–	–	–
Child	–	1 (3)	–	–	–
Friend	–	1 (3)	–	–	–
* Missing*	–	–	–	–	–
Profession					
Occupational therapist	–	–	12 (16)	–	–
Physiotherapist	–	–	13 (18)	–	–
Social worker	–	–	2 (3)	–	–
Physician	–	–	9 (12)	–	–
Psychologist	–	–	1 (1)	–	–
Rehab assistant	–	–	2 (3)	–	–
Nurse	–	–	14 (19)	–	–
Assistant nurse	–	–	21 (32)	–	–
* Missing*	–	–	4	–	–

The inputs (such as issues and statements) provided by the respondents were first checked to ascertain that they addressed potentially unanswered question. If, for example, the inputs explored needs or involved a quest for information about rules and regulations they were considered beyond the scope of the research. In a content analysis, similar inputs were merged to formulate questions that, in turn, were then categorised according to content.

### Literature review

A literature review was conducted, primarily using PubMed, to determine whether every question was answered or unanswered by the current literature. In line with the JLA guidelines, only reviews were included, but we allowed up to 5 years of reviews (instead of the three recommended by JLA) so as not to miss any relevant ones. We used two overall blocks of search terms, one for our target group and one for reviews. For each question, those blocks were supplemented with specific blocks derived from Medical Subject Headings (MeSH) terms and their associated entry terms (Appendix 1, sheet 2). Several questions were of a kind that medical journals may not answer; for these, both CINAHL and PsycINFO were also used to assess the status of evidence (Appendix 1, sheet 3).

All reviews found through every search on every question were assessed in Rayyan, an online and mobile browser for literature review [16]. The assessors were a clinician, a researcher, a linguist and a person with lived experience. Initially, two of the assessors investigated whether the question was answered or unanswered by the literature, and if they did not agree a third person assisted and/or we discussed it in the research team.

Several reviews partially answered the questions—that is, provided initial evidence, which was noted (Appendix 1, sheets 2 and 3). This step, which goes beyond the JLA guidelines, did not affect whether the questions were included in the ensuing steps.

### Interim prioritisation survey

An online survey, using the Swedish University Computer Network (SUNET) Survey tool, was conducted in May and June 2022 among people living with SCI, relatives of people with SCI as well as health professionals and personal care assistants working with people with SCI were included. Respondents were recruited in social media, clinics and networks all over Sweden. The survey was open for 7 weeks. In total, 272 people responded. Table 2 shows the respondents’ personal characteristics.

**Table 2:** Personal characteristics of respondents in the interim prioritisation survey.

	People living with SCI	Relatives	Health professionals	Personal care attendants	Total
	*n* = 156 (57)	*n* = 26 (10)	*n* = 83 (31)	*n* = 7 (3)	*n* = 272
Gender					
Male	75 (48)	6 (23)	18 (22)	1 (14)	100 (39)
Female	81 (52)	20 (77)	64 (78)	6 (86)	171 (63)
* Missing*	–	–	1	–	1
Age, years					
Mean (SD)	53 (13)	53 (14)	47 (12)	49 (13)	51 (13)
Median (range)	52 (18–79)	52 (31–81)	48 (23–87)	55 (30–61)	51 (18–87)
<30	11(7)	–	5 (5)	–	17 (6)
31–45	27 (17)	7 (28)	33 (40)	1 (14)	68 (25)
46–60	73 (47)	10 (39)	34 (41)	1 (14)	121 (44)
60–75	39 (25)	7 (28)	10 (12)	4 (57)	57 (21)
>75	4(3)	1 (1)	1(1)	1 (14)	6 (2)
* Missing*	2	1	–	–	3
Municipality group					
Large cities and nearby municipalities	66 (43)	12 (48)	38 (47)	3 (50)	119 (45)
Medium-sized towns and nearby municipalities	50 (32)	7 (28)	37 (46)	1 (17)	96 (36)
Smaller towns/urban areas and rural municipalities	37 (24)	6 (24)	6 (7)	2 (33)	51 (19)
* Missing*	3	1	2	1	7
Time post injury (TPI)					
Mean (SD)	21 (16)	–	–	–	–
Median (range)	16 (1–61)	–	–	–	–
1–5	25 (17)	–	–	–	–
6–10	27 (18)	–	–	–	–
11–15	21 (14)	–	–	–	–
16–20	10 (7)	–	–	–	–
21–25	7 (5)	–	–	–	–
26–30	15 (10)	–	–	–	–
>30	42 (29)		–	–	–
* Missing*	9	–	–	–	–
Cause of injury					
Traumatic	103 (70)	–	–	–	–
Non-traumatic	44 (30)	–	–	–	–
* Missing*	9	–	–	–	–
Type of injury					
Complete	68 (45)	–	–	–	–
Incomplete	82 (55)	–	–	–	–
* Missing*	6	–	–	–	–
Tetraplegia	56 (38)	–	–	–	–
Paraplegia	149 (62)	–	–	–	–
* Missing*	9	–	–	–	–
Relation					
Partner	–	14 (54)	–	–	–
Parent	–	8 (31)	–	–	–
Sibling	–	3 (12)	–	–	–
Child	–	–	–	–	–
Friend	–	–	–	–	–
* Missing*	–	–	–	–	–
Profession					
Occupational therapist	–	–	19 (25)	–	–
Physiotherapist	–	–	17 (22)	–	–
Social worker	–	–	2 (3)	–	–
Physician	–	–	11 (14)	–	–
Psychologist	–	–	1 (1)	–	–
Rehab assistant	–	–	1 (1)	–	–
Nurse	–	–	12 (16)	–	–
Assistant nurse	–	–	14 (18)	–	–
* Missing*	–	–	6	–	–

The survey was in two parts. In the first, the respondents were asked to tick all the questions they considered important, whereupon a shortlist was produced. In the second part, respondents were asked to select the ten questions they deemed most important.

A frequency table was drawn up to show how often questions were selected, and the questions were then ranked in terms of priority. The rankings were analysed according to subgroup (target group, gender of people with SCI, cause and type of injury) to make sure no high-priority question was eliminated ahead of the final prioritisation workshop. The expert group advised the research group on the selection of questions to proceed to the final prioritisation workshop.

### Final prioritisation workshop

The final prioritisation workshop was organised as a hybrid event with 14 participants in person and nine online. Participants were recruited in social media, clinics, and networks all over Sweden. In total, the participants comprised ten people living with SCI, eight health professionals, three people living with SCI who also worked as health professionals and two relatives who also worked as health professionals. The gender balance among those living with SCI was nine men and 14 women, their mean age was 54 years (SD 11.41, median 52, range 35–76) and the mean time post injury for the people living with SCI was 27 (SD, 12.14, median 29.5, range 12–52). The participants also included two representatives from the expert group.

From the interim prioritisation survey, a shortlist of the highest ranked questions was drawn up and sent to the participants in advance. Before the workshop, they were asked to familiarise and reflect on the questions and to compile their own priority ranking.

After an initial introduction, the participants were divided into four groups. The intention was to achieve a balance among the target groups and in terms of geographical distribution in each group, but this was not fully implemented owing to the hybrid format. At this stage, one group comprised participants online, while three groups comprised participants in person. In the first small-group discission, all the participants presented their top three and bottom three priorities in order to identify similarities and differences among the individuals’ rankings. This was followed by merging of individual priorities to form an agreed initial ranking of questions from each individual small group. All the groups’ rankings were then weighted and used to draw up a master ranking list. (Details of the ranking procedure may be found in the *JLA Guidebook* [15].)

In the second small-group session, new groups were formed so that participants were mostly placed with people who were new to them. In the new groups, participants were asked to discuss the rankings and propose changes. Here again, the new ranking of questions from the session was recorded and weighted to produce a further ranking list.

The second ranking list was discussed in a large group, with all participants needing to agree on a final list of priorities.

The workshop facilitators had no connection with SCI or the JLA method, but were trained by our JLA adviser in line with their programme. Observers from the research team provided supplementary technological support for the online group(s) and, where necessary, information about the PSP process in the group discussions.

## Results

The results presented here are, first, those regarding the questions identified and the literature review. Subsequently, Fig. 1 also illustrates the numerous inputs from the initial survey that had been processed to draw up the initial 57 questions and then the top ten priorities.

### Questions identified

The respondents provided 431 inputs (such as issues and statements), addressing questions they found difficult to answer, although 128 (such as expressing needs or requesting knowledge about rules and regulations) were classified as beyond the scope stipulated. Accordingly, 303 inputs were analysed; these related predominantly to *Mental health and relationships* and *Ageing with SCI*, followed by *Physical health*. However, when similar inputs were merged to formulate questions, it was a matter of equalising the numbers in all categories except *Community support and personal care assistance*. Table 3 presents the numbers of statements in the various categories and the numbers of questions remaining after similar statements were merged to form questions (*n* = 57) in each category.

**Table 3:** Summary of numbers of statements, questions and priorities, by category.

	No. (%) of statements (*n* = 303)	No. (%) of questions (*n* = 57)	No. (%) of questions, final list (*n* = 24)	No. (%) of top ten priorities
Physical health	64 (21)	11 (19)	9 (38)	4 (40)
Body functions	21 (7)	10 (18)	3 (13)	1 (10)
Mental health and relationships	87 (29)	13 (22)	4 (17)	2 (20)
Community support and personal care assistance	5 (2)	4 (7)	2 (8)	1 (10)
Care and rehabilitation	44 (15)	10 (18)	4 (17)	2 (20)
Ageing with SCI	81 (27)	9 (16)	2 (8)	–

### Evidence

The number of hits for every question in the literature search may be found in Appendix 1, sheets 2 and 3. These spreadsheets also present the references for initial evidence. Based on the existing literature, one question was deemed to have been answered:

1. How common is Sleep Apnea Syndrome among people living with tetraplegia [17–19]?

Consequently, 56 questions were unanswered and proceeded to the prioritisation phase.

### Interim prioritisation

In general, the top ten for the full cohort mostly coincided with the top ten for the subgroups as well. There were, however, three notable exceptions. The question *How can relatives give the best support to persons living with SCI?* was ranked fifth for the subgroup with relatives but 12–39th (out of 56) for the other subgroups. Two questions—*For people with spinal cord injury, what kinds of help and treatment are effective in preventing and remedying breathing problems?* and *For people with spinal cord injury, what effects do compression stockings have?*—ranked high (1st and 5th out of 32, respectively) for personal care attendants but not for other subgroups. However, only five personal care attendants responded in the interim survey, and their rankings were therefore addressed in the expert group.

To decide on where to draw the line for the number of questions to be carried forward to the final prioritisation workshop, the expert group were consulted. They were shown the list, including the rankings for all subgroups (Appendix 2), and the decision was then taken. Inclusion of the six top-ranking questions from all subgroups except personal care attendants yielded a total of 20 questions. In addition to these, the question most important to the personal care attendants (*For people with spinal cord injury, what kinds of help and treatment are effective in preventing and remedying breathing problems?*) was included. So, too, were three other questions to balance the number of initial inputs and the categories: *For people with spinal cord injury, what complications and problems are common in old age? After spinal cord injury, how do various technical aids affect people’s ability to become or be independent?* and *In what ways can people with spinal cord injury masturbate and have sex, and what kinds of support and treatment in terms of their sexuality are effective?*

### Final prioritisation

Table 4 presents the top ten priorities, while priorities 11–24 can be found in Appendix 3. As shown in Tables 3 and 4, four of the top ten priorities address *Physical health*, while none of them address *Ageing with SCI*. Interventions to minimise complications related to ageing rank 12th in the final priority list.

**Table 4:** Top ten priorities.

Rank	Category	Question
1	Care and rehabilitation	What kinds of specialist care, from the emergency stage to rehabilitation and lifelong follow-up, should be available?
2	Physical health	What kinds of support and treatment are effective in preventing, relieving and managing pain?
3	Physical health	What kinds of support and treatment are effective in preventing, treating and managing gastrointestinal problems?
4	Care and rehabilitation	How can the care services lacking specialist expertise in spinal cord injuries best respond to people with these injuries and meet their needs? (For example, local health centres and other primary care facilities, or the care services in other specialist areas).
5	Mental health and relationships	What kinds of help and treatment are effective in helping people to cope with their new life situation, and to find ways of enjoying life, after spinal cord injury?
6	Physical health	What kinds of help and treatment are effective in preventing and remedying complications?
7	Physical health	What kinds of help and treatment are effective in preventing and remedying breathing problems?
8	Mental health and relationships	What kinds of support do close relatives of people with spinal cord injury need?
9	Community support and personal care assistance	The Swedish Act concerning support and service for people with certain functional impairments (LSS) applies to the under-65s. What are the implications of this restriction for people with spinal cord injury when they turn 65, and for those who get spinal cord injury in old age?
10	Body function	What kinds of treatment are effective in improving the healing (regeneration) ability of damaged nerves?

## Discussion

Following the comprehensive and inclusive PSP process from JLA, 24 prioritised questions were ranked in the final prioritisation workshop, providing a top ten list of research priorities for SCI. The ten highest priorities include issues at individual level relating to *Physical health*, *Body functions* and *Mental health*, and at societal level including *Care and rehabilitation organisation*, and *Community support and personal care assistance*.

With the need to move research out of hospitals and also include quality-of-life aspects for people living with SCI [5–10], it was somewhat surprising that half of our top ten priorities included typical medical care issues. Priorities included categories like *Physical health* and *Body functions*. On the other hand, for those living with SCI who are suffering from pain and secondary complications, appreciating their social life, relationships, leisure activities and so forth may be difficult. Similarly, problems associated with physical impairment and a sense of loss have a negative impact on quality of life for people living with SCI [7]. However, it is therefore important to balance such complications by developing new values and perspectives, reconstructing sound self-esteem and attaining a sense of biographical continuity [7]. In line with this, it was argued in a recent systematic review that psychosocial care should be considered just as important as physical rehabilitation, and dynamic interaction among physical, psychological and social factors was emphasised [8].

The balance among physical, psychosocial and social factors in SCI rehabilitation is reasonably reflected in the highest priority in this study: *What kinds of specialist care, from the emergency stage to rehabilitation and lifelong follow-up, should be available?* This question was given a high ranking in the interim prioritisation by all subgroups of people living with SCI, but ranked only 38th by health professionals (Appendix 2). In the final workshop, however, there was little discussion of this question and a consensus on its importance was evident. One reason for the significance of this question may be related to reorganisation of SCI rehabilitation in Sweden. From 2023, four national specialist centres will be established to secure multidisciplinary and multiprofessional expertise during initial rehabilitation [20]. Although these political decisions have already been taken, our top-priority question may involve monitoring whether creation of the national specialist centres leads to the expected outcomes. The question of monitoring the national specialist centres should also, of course, be considered along with question four on the priority list: *How can the care services lacking specialist expertise in spinal cord injuries best respond to people with these injuries and meet their needs?* The new specialist centres’ main responsibility is the patients’ initial rehabilitation, but living with SCI requires more contact with the health care system than this. How these non-specialist institutions best respond to people with SCI was also defined as a priority issue.

In the initial phase of issue identification, 27% of the issues selected concerned ageing with SCI, but none of the top ten priorities addressed this topic. Our literature review found one study providing partial or initial evidence for several of the questions, but the authors encourage more studies “to better comprehend the complex relationship between ageing and spinal cord injury and its effects on people’s quality of life” [21]. A recent Swedish study of ageing with SCI shows that older adults with SCI may have a high overall quality of life but simultaneously suffer from a sense of physical and social limitations and injury-related problems [22]. Thus, although there was no specific priority for ageing with SCI, one must remember to include older people and their specific needs, just as other subgroups’ needs should be considered. For instance, several of the 56 questions also specifically concerned incomplete or non-traumatic injuries and women’s health. These are questions that may be assigned high priority in the subgroup but are too specific to reach the top ten for the general SCI population. To highlight these aspects, all 56 questions are listed in Appendix 2.

In line with the previous PSP for SCI [13], the top ten priorities included cure and stem cell research. In this PSP, it should be emphasised that for the workshop participants, this question concerned not only cure but also ways of reducing risks of nerve damage and promoting healing in the acute phase, to optimise the scope for maximising functional recovery. Two recent reviews were identified in our literature review as providing initial or partial evidence for the question *For people with spinal cord injury, how reliably and how soon after the injury can their attainable level of functional capacity be predicted?* The initial American Spinal Injury Association (ASIA) Impairment Scale score has proved to be a strong predictor of both neurological outcome [23] and chronic functional outcome [24]. Furthermore, surgical and specialised functional rehabilitation measures are also emphasised as the strongest predictors of chronic functional impairment. However, future studies are urged to consider in addition, first, management by a specialist multidisciplinary team during emergency care and, second, the intensity and degree of patient participation in rehabilitation therapies and nursing care during the acute phase [24]. The latter two aspects may both fit well together with the tenth prioritised question: *What kinds of treatment are effective in improving the healing (regeneration) ability of damaged nerves?*

In the literature review, only one of the 56 questions covering issues important to people living with SCI was answered. It may be considered discouraging that research has not yet achieved more. Although reviews from the past 5 years (not three as recommended by JLA) were allowed, the requirement of what evidence is needed to deem a question “answered” was rigorous. The reason for this is to minimise the risk of questions not being fully answered by current research. However, since we reviewed a large volume of literature, we also found several initial or partial items of evidence (see Appendix 1). For questions supported by initial or partial evidence, we would encourage researchers to complement existing studies so that the combined studies clarify the issues that people with SCI find difficult to resolve.

In any interpretation of the findings and priorities presented here, there are methodological issues to be considered. First, despite good intentions and several strategic steps in recruitment, representation is questionable at some stages. For example, the final workshop did not include people living with SCI that was incurred recently (time post injury was between 12 and 52 years). Another example is that throughout all the stages, very few personal care attendants have taken part. Second, the inputs in the first survey were of very different kinds, such as specific medical questions, as opposed to against brief statements about “ageing” or “sexual health”. Consequently, the questions processed were at various levels and were difficult to compare and rank. Third, during the final prioritisation, it was evident that focusing on the questions ranked from 8th to 12th—that is, whether these questions should be included in the top ten priorities or not—was most important for the participants, while the priorities ranked in the first seven were discussed less. This may be due to a stronger consensus in the initial group phases of the prioritisation workshop, but it also reflects the relevance of a list of precisely ten top priorities. Again, we emphasise that the full priority list is provided in Appendix 3, and all 57 important questions may be found in Appendix 2.

To conclude, this work has studied questions that people living with SCI, relatives of people with SCI as well as health professionals and personal care assistants working with people with SCI in Sweden find hard to get answered. Based on the comprehensive, inclusive PSP process from JLA, a top ten list of research priorities for SCI has been drawn up. We support the claim of the previous PSP in SCI in the UK to have issued a call to action to SCI researchers, urging them to devise appropriate studies for achieving optimal scientific progress in the issues that matter most to people with SCI [13]. In particular, the top-priority questions for people with SCI in Sweden concern specialist SCI care and rehabilitation, and a number of questions addressing physical health follow. However, the full list of key questions broadens the scope of the study to also include the topics of *Mental health*, *Ageing with SCI*, *Community support and personal care assistance*, and *Body functions*.

### Supplementary information

Appendix 1

Appendix 2

Appendix 3

## Data Availability

The data generated or analysed in this study are provided in Appendices 1–3 and on the project’s OSF page 10.17605/OSF.IO/73APW.

## Footnotes

1 Under Swedish law [14], personal care attendants can be provided for people who, owing to serious and long-lasting functional impairments, need help to meet their own basic needs.
